# The Relationship Between Personality Traits and Coping Styles Among First-Time and Recurrent Prisoners in Poland

**DOI:** 10.3389/fpsyg.2019.02969

**Published:** 2020-01-14

**Authors:** Magdalena Leszko, Rafał Iwański, Aneta Jarzębińska

**Affiliations:** ^1^Department of Psychology, University of Szczecin, Szczecin, Poland; ^2^Department of Pedagogy, University of Szczecin, Szczecin, Poland

**Keywords:** coping strategies, incarceration, personality traits, prisoners, stress

## Abstract

Individuals with certain personality traits employ adaptive coping strategies. Little research, however, has examined coping strategies among incarcerated individuals. A cross-sectional study was conducted among 465 males who served time in five different prisons in Poland. We examined the relationship between the Big Five personality dimensions and coping styles, and the results demonstrated that neuroticism predicts emotion-oriented coping whereas conscientiousness predicts task-oriented coping strategies. A better understanding of the role of personality traits and its relation to coping strategies may allow for more targeted and effective psychological interventions that will, in turn, improve inmates’ abilities to cope with stress.

## Introduction

The deleterious effects of stress on mental and physical health are well-documented (e.g., Schneiderman et al., 2005; Yaribeygi et al., 2017). Stress is inevitable and every individual experiences some form of stress to which one responds using different strategies in order to reduce negative emotions. Efforts to understand the impact of stress have increasingly focused on the role of coping strategies (e.g., Carver et al., 1989). Although coping has been conceptualized in different ways, many theorists emphasize that coping can be defined as a physiological, behavioral, emotional, and cognitive reaction to changes in environment (Lazarus and Folkman, 1984; Compas et al., 2001; Beutler et al., 2003). Even though coping is a dynamic process that fluctuates over time in response to changing demands and appraisals of the situation, most individuals respond to stress in a consistent manner and apply one style over a variety of situations (Endler, 2009).

Responses to stressful events or conditions are typically broken into three main coping styles: task-oriented, emotion-oriented, and avoidant-oriented coping (Endler and Parker, 1990). Task-oriented coping refers to a response that aims at reducing or removing the source of stress by taking action to solve the problem or altering the situation if the problem cannot be removed. Emotion-oriented coping is self-oriented and includes a variety of behaviors which aim at diminishing distress caused by the stressors. Such behaviors may include the expression of negative emotions or seeking emotional support. The avoidance-oriented coping style can take a form of activities that allow people to emotionally or physically distance themselves from the stressor by, for example, denying, engaging in distracting behavior (e.g., performing a different task) or employing maladaptive behaviors such as alcohol or drug abuse. Studies have shown that inadequate coping skills are related to an increased likelihood of both mental disorders and suicidal behavior (Shneidman, 1992; Horwitz et al., 2011), whereas adaptive coping (e.g., task-oriented coping) decreases symptoms of adverse psychological reactions to stress (e.g., Antoni et al., 2001; Connor-Smith and Compas, 2004). Thus, it is important to understand what factors affect coping strategies.

Research findings indicate that how an individual copes with various problems may be influenced by personality traits (Costa et al., 1996). It has been empirically established that the “Big Five” personality traits of neuroticism (vs. emotional stability), extraversion (vs. introversion), openness to experience (vs. closeness to experience), conscientiousness (vs. lack of direction), and agreeableness (vs. antagonism), are associated with one’s coping strategy selection (Kardum and Krapić, 2001; Connor-Smith and Flachsbart, 2007). For example, individuals high on the neuroticism dimension tend to experience more intense emotional and physiological reaction to stress and evaluate stressors as highly threatening (Costa and McCrae, 1987; Penley and Tomaka, 2002). In order to decrease negative affect such as anxiety, sadness, or depression, these individuals rely on emotion-oriented coping or disengagement. That allows them a temporary relief from experiencing unpleasant emotions but prevents them from engagement in solving the problem (Carver and Connor-Smith, 2010). Another strategy used by individuals high on neuroticism is to seek social support for emotional reasons such as venting (Vollrath et al., 1998). Extraversion has consistently been shown to be moderately correlated with employing problem-solving strategies (Vollrath, 2001). Individuals high on extraversion are also more likely to seek instrumental support (asking for advice or information) (e.g., Russell et al., 1997; Asendorf and Wilpers, 1998). Individuals with high levels of conscientiousness are more self-disciplined, goal-directed (Costa and McCrae, 1992), tend to be careful about taking action that could possibly damage their reputation (Caligiuri, 2000) but focus on organizing plans that will alleviate the impact of a stressful situation (Ferguson, 2001). Findings on conscientiousness demonstrate that this personality trait is associated with distraction or cognitive restructuring (e.g., evaluating and modifying negative thoughts, shifting attention from the negative toward positive thoughts or activities) (Vollrath and Torgersen, 2000; Connor-Smith and Flachsbart, 2007). With regard to agreeableness, most studies have found a significant positive correlation between this personality trait and seeking social support and cognitive restructuring (Penley and Tomaka, 2002; Carver and Connor-Smith, 2010). Finally, high scores on openness to experience have demonstrated an association with positive reappraisal, seeking information, and problem solving, but a negative association with avoidance coping (Rothbart and Bates, 1998; Penley and Tomaka, 2002; Carver and Connor-Smith, 2010).

Prior research has confirmed that personality predicts coping styles but understanding this link has been hindered by an almost exclusive reliance on studies conducted on convenience samples. For example, in their meta-analysis of the relationship between personality and coping, Connor-Smith and Flachsbart (2007) reviewed 165 studies and most of them relied on students. While these studies provide evidence for the significant role of personality, they are limited to samples that experience the stresses of everyday life. Researchers emphasize that the relationship between personality and coping might be influenced by the context in which the stressor occurs and that the relationship is stronger in samples that face physically or mentally demanding situations for an extended period of time (DeLongis and Holtzman, 2005; Connor-Smith and Flachsbart, 2007). Among chronic stress-related conditions, incarceration may have a profound impact on the selection of coping strategies (Brown and Ireland, 2006; Massoglia, 2008).

Serving time in prison can be especially stressful as it affects different domains of life and significantly limits individuals’ resources to cope with stress. Inmates have to adapt to a new environment and experience the negative aspects of incarceration such as a lack of privacy, loss of freedom, isolation, and limited contact with family and friends (Greve, 2001). In addition, their activities are subject to the prison norms so they have little control over their lives (Haney, 2001; Shammas, 2017). Not surprisingly, the incarceration experience may result in mental health disorders. A recent meta-analysis of studies regarding prisoners’ mental health demonstrates that incarcerated individuals are at an increased risk of all-cause mortality, suicide, self-harm, violence, and victimization (Fazel et al., 2016). Despite exposure to a stressful situation that endures over time, not all incarcerated individuals have a compromised mental well-being (Haney, 2001). Existing evidence of the association between personality traits and coping in general populations indicates that certain personality traits may impact coping strategies and, therefore, account for a protective factor against the short and long-term consequences of stress (Eley et al., 2013). For example, conscientiousness has received a great amount of attention as a protective factor from stress because it has been associated with task-oriented coping. In contrast, emotion-oriented coping is related to a negative affect and poorer adjustment (Bartley and Roesch, 2011).

Given that personality traits can explain some of the variance in the coping strategies in the general population and that appropriate coping-strategy selection may increase an individual’s resilience, whereas maladaptive coping may further increase vulnerability and lead to negative mental health outcomes (Diehl et al., 2012), it is important to examine how personality traits influence the strategies prisoners use to cope with stress.

The purpose of this study is to extend the line of research by testing the relationship between personality traits and coping strategies among 465 incarcerated individuals. Specifically, we examined what personality traits predict certain coping strategies and whether coping strategies differ between first-time and recurrent prisoners. The rationale behind this is that individuals who were in prison earlier were more familiar with the environment. Previous studies found that by the third or fourth month of incarceration, first-time prisoners became assimilated to the regimented and repetitive daily prison routine (Souza and Dhami, 2010). We thus limited our sample to participants who had been in prison for longer than 3 months.

Investigating the coping strategies among incarcerated individuals is important because certain coping strategies might determine whether prisoners interpret their experience in prison as a rehabilitative or detrimental one (Zamble and Porporino, 1990). Adaptation to prison can also affect their post-release functioning (Gerber and Fritsch, 1995).

In addition, the incarcerated population is vastly underrepresented in research. Equitable participation of incarcerated individuals in psychological research is vital because it can advance our knowledge regarding psychological functioning of incarcerated individuals. A better understanding of the link between personality and coping among prisoners may help identify prisoners that tend to use ineffective coping strategies. That in turn, will help design effective programs aimed at educating prisoners to implement strategies that can be used during their incarceration and after their release, also considered to be a stressful period (Haney, 2001).

This research attempts to address these gaps by investigating the relationship between personality traits and coping strategies using data from 465 individuals serving time in ten Polish prisons. Based on previous research conducted on general populations, it was hypothesized that openness to experience, extraversion, and conscientiousness would be negatively associated with avoidant-oriented coping. Because these three personality traits relate to perceiving events as challenges rather than threats (Carver and Connor-Smith, 2010), we expected that they would be positively associated with task-oriented coping. We also expected that neuroticism would be positively correlated with emotion-oriented coping but negatively with task-oriented coping whereas agreeableness would be positively associated with emotion-oriented coping. On an exploratory basis, the study also investigated whether the status (being first-time vs. recurrent inmate) moderated the associations between personality traits and coping styles by interaction term.

## Materials And Methods

### Procedure

Based on purposive sampling, only convicted prisoners were recruited to participate in this study. The authors obtained permission to conduct the study from the directors of the each of the correctional facilities. The study was conducted in January and February of 2019 by approaches made to five medium-security state prisons in Poland. Potential participants were recruited through announcements made in prisons. Participants were approached through research assistants who explained the conditions of participation (including confidentiality and a right to withdraw from the study). Upon giving their informed consent, participants completed questionnaires in a group setting. All questionnaires were completed under the supervision of two of the authors. There were no incentives for participation. The study was conducted according to the criteria set by the declaration of Helsinki and was approved by the Institutional Review Board of University of Szczecin. We used descriptive statistics, Pearson correlation coefficient, and multiple linear regression analysis of the data. The effect sizes were estimated with η^2^. *A priori* power analysis conducted in G^∗^Power (Faul et al., 2009) for the nested regression models with six predictors on the first block and five more predictors (defined as interaction terms) on the second block revealed that interaction term should explain (defined as *R*^2^ change) more than 0.07 of outcome variable variance to achieve sufficient power (1-beta = 0.80) with alpha set at α = 0.05 and sample size *N* = 465 and expected total *R*^2^ on 0.15. Data analysis was conducted using SPSS 23.0.

### Participants

The total sample included 465 incarcerated individuals aged from 18 to 68 years (*M* = 35.1; *SD* = 9.5) serving their time in Polish prisons who participated in the study on a voluntary basis. The participants were divided into two groups: (a) first-time male inmates (*n* = 201) and (b) recurrent male inmates (*n* = 265). One hundred percent of the participants in this sample were Caucasian. All respondents were Polish citizens. Participants also met the following criteria: (a) being incarcerated for more than 3 months, (b) lacking a diagnosis of mental illness (c) possessing the ability to read and write. The researchers excluded from the analyses 12 participants who did not complete the questionnaires.

### Measures

#### NEO Five Factor Inventory (NEO-FFI)

NEO Five Factor Inventory is a 60-item self-report instrument used to measure individual differences in personality factors, with 12 items for each factor. The NEO FFI includes self-descriptive statements that participants respond to using a 1 (strongly disagree) to 5 (strongly agree) Likert-type scale. Scores for each domain were calculated by summing up the 12 item responses. A total of 28 NEO FFI items are reverse-worded (Costa and McCrae, 1992). Internal consistency of the subscales (Cronbach’s alpha) was satisfactory and ranged from 0.60 to 0.83.

#### Coping Inventory for Stressful Situations (CISS)

The CISS is a 48-item, self-report measure of coping strategies to which subjects respond on a five-point Likert scale ranging from 1 (not at all) to 5 (very much). The scale includes three dimensions of coping: avoidance-oriented, emotion-oriented, and task-oriented coping. Each coping style is measured by 16 items. The avoidance coping is divided into two dimensions: distraction (eight items) and social diversion (eight items) (Endler and Parker, 1990). Each subscale consists of 16 statements and the result obtained for each of them ranges from 16 to 80 points. The reliability of the scale, indicated by Cronbach’s alpha, ranged from 0.78 to 0.90.

The questionnaires also included questions about demographics (e.g., education level, age, marital status, and employment history).

## Results

Means and standard deviations for all variables stratified by inmates status (first-time vs. recurrent) are presented in Table 1. A total of 465 participants were included in our study.

**TABLE 1:** Sample characteristics.

**Variables and categories**	**First-time inmates (*n* = 200)**	**Recurrent inmates (*n* = 265)**	**Overall sample (*N* = 465)**
	
	***M* (*SD*) or% (*N*)**	***M* (*SD*) or% (*N*)**	***M* (*SD*) or% (N)**
Age (range 18–68)	32.9 (10.1)	36.7 (8.7)	35.1 (9.5)
**Respondent’s Education**			
Less than high school	65%(129)	76%(203)	71%(332)
High school	29%(58)	23%(60)	25%(118)
College degree	6%(13)	1%(2)	4%(15)
**Five-factor model**			
Extraversion	39.3 (5.9)	38.4 (5.9)	38.8 (5.9)
Neuroticism	33.3 (8.6)	33.8 (8.6)	33.6 (8.6)
Openness to Experience	36.4 (4.5)	36.7 (5.0)	36.5 (4.8)
Conscientiousness	47.2 (7.7)	45.9 (8.0)	46.5 (7.9)
Agreeableness	41.5 (6.4)	40.3 (6.2)	40.8 (6.3)
**Coping strategies**			
Task-oriented coping	58.8 (9.4)	58.04 (8.8)	58.4 (9.1)
Emotion-oriented coping	45.03 (10.2)	46.6 (10.1)	45.9 (10.2)
Avoidance coping	50.7 (9.6)	51.7 (9.3)	51.3 (9.4)

An independent samples *t*-test was conducted to compare the scores between fist-time and recurrent inmates. First-time inmates had higher agreeableness (*M* = 41.5, *SD* = 6.4) than recurrent inmates (*M* = 40.3, *SD* = 6.2). This difference was significant, [*t*(463) = 2.03, *p* < 0.05, *d* = 0.19, 95% CI = (0.05, 0.32)]. However, the magnitude of the difference in the means was very small (eta squared = 0.001). First-time inmates were also significantly older that recurrent inmates (*M* = 32.9, *SD* = 10.01 vs. *M* = 36.7, *SD* = 8.7), [*t*(463) = −4.37, *p* < 0.001, *d* = −0.41, 95% CI = (−0.54, −0.27)]. The magnitude of the differences in the means was small (eta squared = 0.04). No significant differences were found for other personality traits nor coping styles.

### Correlations Between Big Five Personality Traits and Coping Styles

To examine the association between personality traits and coping styles, Pearson’s correlations were obtained for the whole sample (Table 2). In a supplementary analysis we stratified the correlation analyses by imprisonment status (first-time inmates vs. recurrent inmates) (see Supplementary  Material). The results showed that there was a significant relationship between age and agreeableness (*r* = 0.12, *p* < 0.01), educational attainment (*r* = −0.19, *p* < 0.01), and avoidance coping (*r* = −0.15, *p* < 0.01). Task-oriented coping was associated with all personality traits. It was positively associated with extraversion (*r* = 0.24, *p* < 0.01), openness to experience (*r* = 0.13, *p* < 0.01), agreeableness (*r* = 0.16, *p* < 0.01), and conscientiousness (*r* = 0.49, *p* < 0.01) but negatively associated with neuroticism (*r* = −0.15, *p* < 0.01).

**TABLE 2:** Correlations between personality traits and coping styles (*N* = 465).

	**1**	**2**	**3**	**4**	**5**	**6**	**7**	**8**	**9**
(1) Age									
(2) Education	–0.19^∗∗^								
(3) Extraversion	–0.09	0.16^∗∗^							
(4) Neuroticism	–0.04	–0.14^∗∗^	–0.36^∗∗^						
(5) Openness	0.09	0.12^∗∗^	0.08	–0.03					
(6) Agreeableness	0.12^∗∗^	0.05	0.24^∗∗^	–0.28^∗∗^	–0.01				
(7) Conscientiousness	0.02	0.08	0.36^∗∗^	–0.39^∗∗^	0.06	0.45^∗∗^			
(8) Task-oriented coping	0.02	0.15^∗∗^	0.24^∗∗^	–0.15^∗∗^	0.13^∗∗^	0.16^∗∗^	0.49^∗∗^		
(9) Emotion-oriented coping	–0.02	–0.11^∗∗^	–0.18^∗∗^	0.62^∗∗^	–0.08	–0.18^∗∗^	–0.24^∗∗^	0.06	
(10) Avoidance coping	–0.15^∗∗^	–0.15^∗∗^	0.23^∗∗^	0.09^∗^	–0.05	–0.05	–0.01	0.16^∗∗^	0.27^∗∗^

*^∗∗^Correlation is significant at the 0.01 level (2-tailed). ^∗^Correlation is significant at the 0.05 level (2-tailed).*

Emotion-oriented coping was negatively associated with extraversion (*r* = −0.18, *p* < 0.01), agreeableness (*r* = −0.18, *p* < 0.01), and conscientiousness (*r* = −0.24, *p* < 0.01). This coping style was positively associated with neuroticism (*r* = 0.62, *p* < 0.01). No significant associations were found in between emotion-oriented coping and openness to experience. Avoidance coping style was negatively associated with age (*r* = −0.15, *p* < 0.01) but positively associated with extraversion (*r* = 0.23, *p* < 0.01) and neuroticism (*r* = 0.09, *p* < 0.05).

We further investigated if there are significant differences in the correlation between first-time and recurrent inmates. Therefore, Pearson correlations between coping styles and each personality score were calculated, separately for each group. All correlation coefficients were converted using Fisher’s *r*- to -*z* transformation to achieve *z* values with an approximately normal distribution and the related 95% confidence interval. The results of Fisher’s z transformation are presented in Supplementary Material. Results of the analysis revealed no significant differences between correlations obtained in both samples. There were no significant differences in correlations between first-time and recurrent inmates.

### Status Differences: Exploratory Analysis

In order to verify the hypothesis that status (being first-time vs. recurrent inmate) moderates the effect of personality on coping styles we conducted a moderation analysis. We (a) estimated multiple linear regression using bootstrap resampling where the outcome variable (each of three coping styles) was predicted by moderator (status) and the main predictors (personality traits), (b) calculated indicators of interaction between moderator and main predictors as a multiplication of their scores centered around the mean, and (c) estimated the multiple linear regression model using bootstrap resampling method where outcome variable by moderator and main predictors and their interactions.

#### Task-Oriented Coping

The results revealed that when predicting the coping styles, the model was significant [*R*^2^ = 0.260; *F*(5,458) = 23.816; *p* < 0.001]. However, the results showed that the interaction term did not increase the *R*^2^ significantly [Δ*R*^2^ = 0.006; Δ*F*(4,453) = 1.083, *p* = 0.115]. Personality traits predicted coping styles orientation similarly in population of first-time prisoners and recurrent inmates. Therefore the model predicting task-orientation coping style was estimated for both groups jointly. The results of the regression for both groups indicated that conscientiousness is the strongest predictor of task-oriented coping style (*b* = 0.58^∗∗∗^, *r*^2^ = 0.19) and explains 19% of the variance (see Table 3).

**TABLE 3:** Results of the regression analysis predicting task-oriented coping.

**Predictor**	***b***	***SE(b)***	**β**	***t*(456)**	***p***	**Partial *R*^2^**
Status	–0.148	0.752	–0.008	–0.196	0.844	0.000
Neuroticism	0.068	0.049	0.064	1.378	0.168	0.004
Extraversion	0.138	0.062	0.090	2.227	0.026	0.011
Openness	0.181	0.072	0.096	2.527	0.011	0.014
Agreeableness	–0.101	0.070	–0.071	–1.448	0.148	0.005
Consciousness	0.578	0.056	0.505	10.388	0.001	0.191

#### Emotion-Oriented Coping

The results of regression analysis predicting emotion-oriented coping indicated that the model was significant [*R*^2^ = 0.260; *F*(5,458) = 23.816; *p* < 0.001], but the interaction effect did not increase the *R*^2^ significantly [Δ*R*^2^ = 0.006; Δ*F*(4,453) = 1.083, *p* = 0.115]. Only neuroticism significantly predicted this coping strategy (*b* = 0.75; *r*^2^ = 0.34) (see Table 4).

**TABLE 4:** Results of the regression analysis predicting emotion-oriented coping.

**Predictor**	***b***	***SE(b)***	**β**	***t*(456)**	***p***	**Partial *R*^2^**
Status	1.256	0.708	0.061	1.775	0.076	0.007
Neuroticism	0.750	0.049	0.628	15.349	0.001	0.341
Extraversion	0.100	0.07	0.058	1.429	0.153	0.005
Openness	−0.141	0.078	−0.067	−1.815	0.070	0.007
Agreeableness	−0.029	0.075	−0.018	−0.389	0.697	0.000
Consciousness	0.008	0.058	0.006	0.131	0.896	0.000

#### Avoidance Coping

The results revealed significant, but not large in sense of effect size *R*^2^ [*R*^2^ = 0.100, *F*(5,458) = 9.160, *p* = 0.001] effect of personality traits on avoidance coping. The inclusion of the interaction term was not significant [Δ*R*^2^ = 0.010, Δ*F*(4,453) = 1.272, *p* = 0.079]. Conscientiousness was a significant predictor of avoidance coping style (*b* = −0.169, *r*^2^ = 0.01) (see Table 5).

**TABLE 5:** Results of the regression analysis predicting avoidance coping.

**Predictor**	***b***	***SE(b)***	**β**	***t*(456)**	***p***	**Partial *R*^2^**
Status	1.234	0.854	0.065	1.444	0.149	0.003
Neuroticism	0.166	0.147	0.075	1.127	0.260	0.008
Extraversion	0.135	0.157	0.042	0.857	0.391	0.006
Openness	0.03	0.197	0.008	0.153	0.879	0.001
Agreeableness	0.175	0.161	0.059	1.087	0.277	0.008
Consciousness	−0.169	0.101	−0.07	−1.673	0.095	0.008

## Discussion

Personality plays an important role in coping with stress. People respond to stress by engaging in various behaviors in order to decrease experiencing unpleasant emotions. Most of the previous research on the relationship between personality traits and coping relied on employing convenience samples. However, the context in which coping occurs is also very important. Serving time in prison is a stressful, chronic situation that may impact the relationship between personality traits and coping. Therefore, this study focused on the predictive role of personality traits among first-time and recurrent male inmates serving their time in prisons in Poland.

Based on the data from the whole sample, we found that neuroticism was positively associated with emotion-oriented coping but negatively with task-oriented coping. Prisoners who were high on neuroticism tended to choose an emotion-oriented coping strategy. These individuals are more likely to cope with stress by getting more angry and tense than individuals whose scores are lower. They also tend to choose self-preoccupation or daydreaming reactions as a way of reducing that stress. As expected, task-oriented coping was positively associated with extraversion, openness to experience, and conscientiousness. We also found that task-oriented coping was positively associated with agreeableness. Avoidance coping style was negatively associated with age but positively associated with extraversion and neuroticism. The results also show that extraversion and neuroticism were associated with avoidance-oriented coping. These men were more likely to engage in activities that would help them to escape feelings of distress, for example, by denial or the use of alcohol. There were no significant differences between associations between first-time and recurrent inmates in terms of the predictive role of personality traits. Results of regression analysis of personality traits on coping styles indicated that neuroticism was a strong predictor of emotion-oriented coping which confirms previous findings in general population (e.g., Connor-Smith and Flachsbart, 2007). Individuals high on conscientiousness tended to use task-oriented coping, whereas these who were low on this personality trait, tended to use avoidance coping. These finding are also in line with previous findings emphasizing the role of conscientiousness in choosing an effective coping style (Carver and Connor-Smith, 2010).

The study demonstrates that personality traits play an important role in predicting coping strategies and adds to the literature by comparing first-time and recurrent inmates. Previous research has indicated that the magnitude of correlations between personality and coping may vary across samples (Connor-Smith and Flachsbart, 2007), emphasizing the need to investigate the relationship in different contexts and among more diverse populations, including prisoners. Conducting research on understudied populations such as prisoners is very important because the prison environment can be a great source of stress and an adjustment to its rules can be difficult for many individuals. The failure to adapt may result in poorer mental and physical functioning. Because studies conducted on the general population demonstrated that using emotion-oriented coping may lead to higher levels of depression, depersonalization, and emotional exhaustion (Sears et al., 2000; McWilliams et al., 2003), it is crucial to identify who is more prone to use avoidance or emotion-oriented coping among prisoners.

The findings from this study provide important insights that can be used when designing psychological interventions to teach prisoners how to implement more effective coping strategies. Perhaps some prisoners adapt avoidance-oriented coping (e.g., alcohol use) because they do not know how to cope with unpleasant emotions or they are not familiar with ways of evaluating and modifying negative thoughts. However, when implementing programs designed to teach inmates how to cope with stress, it is important to take into account the role of personality traits.

Personality traits not only predict the choice of one strategy over another, but they also influence the effectiveness of the chosen strategy (DeLongis and Holtzman, 2005). For example, if an individual copes with stress using a strategy that is not optimal for his or her personality, it may result in experiencing negative outcomes (Bolger and Zuckerman, 1995). Gunthert et al. (1999) found that college students who scored high on neuroticism used less-adaptive coping strategies (e.g., hostile reaction) and responded with more distress in reaction to some types of new coping strategies in comparison to students whose scores on neuroticism were low. On the other hand, certain task-oriented activities, such as support seeking, may not require so much diligence and impulse-control and may, therefore, be preferred by individuals who had high neuroticism levels. Teaching prisoners new methods of coping should be tailored to their personality traits and most importantly, their situation and resources. Using task-oriented coping may be effective when an individual perceives that he or she has some control over the situation.

One of the main strengths of this study is that the researchers used data from a large sample of prisoners and employed validated instruments. We compared first-time male inmates’ results to the recurrent male inmates. However, there are several limitations and caution is needed when interpreting the results. The main limitation lies in the cross-sectional nature of the study. Future studies investigating the link between personality traits and coping among prisoners should test whether strategies employed in prisons differ significantly from those who left prison. Another limitation is that the data used in this study was limited to Caucasians, which reflects the demographic distribution of people living in Poland. Prospective research should also employ a more racially diverse sample. The third limitation of this study is that the sample was limited to men only. The reason why women were not included in the analyses is that as the vast majority of incarcerated peoples are men. According to the Prison Service Department, at the end of 2018, there were 64 045 incarcerated individuals (Ministry of Justice – Prison Service Department, 2018). Of those who were incarcerated, only 2 494 were women (3.8%). When designing the study we were planning on including both men and women, however, we were not able to collect data from more than 30 incarcerated women. A small sample size reduces the power of the study and increases the margin of a type II error (Nayak, 2010). Therefore, we decided to limit our study to incarcerated men only. The relationship between personality traits and coping styles might also be different between the two genders; therefore, future studies should test if gender is a moderation of this relationship. There is also a possibility of the volunteer bias which can limit generalizability of our findings. Although the majority of inmates had agreed to participate in our study (95%), previous research demonstrated that there are individual differences in personality characteristics among individuals who agree to participate in a study and those who do not. Specifically, volunteers in general population demonstrated to have higher levels of openness to experience and extraversion in comparison to non-volunteers (Rosenthal and Rosnow, 1975; Sharp et al., 2006). However, for ethical or reasons, we relied on data provided from inmates who volunteered to participate in the study.

Despite the limitations, the current findings are valuable in showing the association between personality traits and coping styles in a large sample facing chronic stress. A better understanding of the role of personality traits and how they are related to coping strategies may allow for more targeted and effective psychological interventions that will in turn improve inmates’ abilities to cope with stress both during their incarceration and upon their release.

## Data Availability Statement

The datasets generated for this study are available on request to the corresponding author.

## Ethics Statement

The studies involving human participants were reviewed and approved by the Ethics Committee of University of Szczecin. Written informed consent for participation was not required for this study in accordance with the national legislation and the institutional requirements.

## Author Contributions

ML contributed to the design of the work, analysis and interpretation of data, applying for IRB approval, and writing the manuscript. RI and AJ contributed to the design of the work and collected data.

## Conflict of Interest

The authors declare that the research was conducted in the absence of any commercial or financial relationships that could be construed as a potential conflict of interest.
