# Supplementary material for: The Relationship Between Personality Traits and Coping Styles Among First-Time and Recurrent Prisoners in Poland
Source: Front Psychol. 2020 Jan 14;10:2969. doi: 10.3389/fpsyg.2019.02969 (PMC6972873; doi:10.3389/fpsyg.2019.02969)
Supplement: Supplementary file 1 [file Table_1.docx]

**Supplementary Materials**

Supplementary Table 1

*Correlation between personality factors and coping with stress indices in sample of first time prisoners (n =200) and recurrent prisoners (n=256)*

|  | First-time inmates (n=200) | | | Recurrent inmates (n=265) | | |
| --- | --- | --- | --- | --- | --- | --- |
|  | Task-oriented coping | Emotion-oriented coping | Avoidance coping | Task-oriented coping | Emotion-oriented coping | Avoidance coping |
| Neuroticism | -.12 (.50) | .65 (.01) | .01 (.99) | -.17 (.05) | .59(.01) | .15 (.10) |
| Extraversion | .20 (.04) | -.16 (.19) | .22 (.02) | .27 (.01) | -.19 (.02) | .24(.01) |
| Openness | .15 (.26) | -.09 (.96) | -.05 (.99) | .12 (.22) | -.07 (.98) | -.05 (.99) |
| Agreeability | .20 (.05) | -.23 (.01) | -.06 (.99) | .13 (.22) | -.13 (.22) | -.02(.99) |
| Consciousness | .53 (.01) | -.22 (.02) | .06 (.99) | .46 (.01) | -.24 (.01) | -.06 (.99) |

*Note:* p-values corresponding to test of null hypotheses that correlation equals zero adjusted for type I error using sequential Holm-Bonferroni procedure are presented in brackets.

Supplementary Table 2

*Summary of the Fisher’s Z tests comparing correlations between personality traits and coping styles in the first-time and recurrent inmates*

|  | Fisher's Z test | | |
| --- | --- | --- | --- |
|  | Task-oriented coping | Emotion-oriented coping | Avoidance coping |
| Neuroticism | Z = .542, *p* = .588,  f = .051,  95% CI [-0.081, 0.183] | Z = 1.035, *p* = .301,  f = .098,  95% CI [-0.034, 0.230] | Z = -1.497, *p* = .134,  f = -.141,  95% CI [-0.273, 0.009] |
| Extraversion | Z = -.786, *p* = .432,  f = -.074,  95% CI [-0.206, 0.058] | Z = .328, *p* = .743,  f = .031,  95% CI [-0.101, 0.163] | Z = -.224, *p* = .823,  f = -.021,  95% CI [-0.153, 0.111] |
| Openness | Z = .324, *p* = .746,  f = .031,  95% CI [-0.101, 0.163] | Z = -.213, *p* = .831,  f = -.02,  95% CI = [-0.152, 0.112] | Z = .000, *p* > .999,  f = .000,  95% CI [-0.132, 0.132] |
| Agreeableness | Z = .763, *p* = .445,  f = .072,  95% CI [-0.060, 0.204] | Z = -1.097, *p* = .273,  f = -.103,  95% CI = [-0.235, 0.029] | Z = -.425, *p* = .671,  f = -.04,  95% CI [-0.172, 0.092] |
| Consciousness | Z = .984, *p* = .325,  f = .093,  95% CI [-.039, 0.225] | Z = .224, *p* = .823,  f = .021,  95% CI [-0.111, 0.153] | Z = 1.274, *p* = .203,  f = .12,  95% CI [-0.012, 0.252] |
